# Brain damage markers neuron-specific enolase (NSE) and S100B in serum in children with Lyme neuroborreliosis—detection and evaluation as prognostic biomarkers for clinical outcome

**DOI:** 10.1007/s10096-022-04460-1

**Published:** 2022-06-06

**Authors:** Sigurdur Arnason, Kesia Molewijk, Anna J. Henningsson, Ivar Tjernberg, Barbro H. Skogman

**Affiliations:** 1 Department of Clinical Science, Intervention and Technology – CLINTEC, Alfred Nobels Allé 8, 141 52 Huddinge, Stockholm Sweden; 2 Department of Pediatric Infectious Diseases, Astrid Lindgren’s Children’s Hospital, Eugeniavägen 23, 171 64 Solna, Stockholm Sweden; 3 Faculty of Health and Medical Sciences, Örebro University, Södra Grev Rosengatan 42 B, S-703 62 Örebro, Sweden; 4 Department of Biomedical and Clinical Sciences, Division of Inflammation and Infection, Linköping University, Linköping, Sweden; 5 National Reference Laboratory for Borrelia and Other Tick-Borne Bacteria, Division of Clinical Microbiology, Laboratory Medicine, Region Jönköping County, Linköping University, Linköping, Sweden; 6 Department of Clinical Microbiology in Linköping, Linköping University, Linköping, Sweden; 7 Department of Clinical Chemistry and Transfusion Medicine, Region Kalmar County, Kalmar, Sweden; 8 Center for Clinical Research Dalarna – Uppsala University, Nissers väg 3, S-791 82 Falun, Sweden; 9 Department of Clinical Science, Intervention and Technology – CLINTEC, Karolinska Institutet, Alfred Nobels Allé 8, S-141 52 Huddinge, Stockholm Sweden

**Keywords:** Lyme neuroborreliosis, S100B, NSE, Clinical outcome, Brain damage markers, Biomarkers

## Abstract

Lyme borreliosis (LB) is the most common tick-borne infection in Europe, with Lyme neuroborreliosis (LNB) its second most frequent clinical manifestation. Prognostic factors for clinical outcomes in LNB have not been identified. Elevated serum levels of the brain damage markers neuron-specific enolase (NSE) and S100 calcium-binding protein B (S100B) have been associated with poor clinical outcomes in other disorders of the central nervous system. The aim of this study is to assess NSE and S100B in serum as prognostic biomarkers for clinical outcomes in paediatric LNB patients. Children evaluated for LNB (*n* = 121) in Sweden were prospectively included during 2010–2014, serum samples were collected on admission, and all children underwent a 2-month follow-up. Patients with pleocytosis and anti-*Borrelia* antibodies in cerebrospinal fluid (CSF) were classified as having LNB (*n* = 61). Controls were age- and gender-matched non-LNB patients (*n* = 60). NSE was elevated in 38/61 (62%) LNB patients and in 31/60 (52%) controls. S100B was elevated in 3/60 (5%) LNB patients and 0/59 (0%) controls. NSE and S100B concentrations did not differ significantly when comparing LNB patients with controls. No differences were found in the concentrations when comparing the clinical recovery of LNB patients at the 2-month follow-up. NSE was detectable in the majority of LNB patients and controls, whereas S100B was detectable in only a few LNB patients and no controls. NSE and S100B in serum cannot be recommended as prognostic biomarkers for clinical outcomes in children with LNB.

## Introduction

Lyme borreliosis (LB) is the most common tick-borne infection in Europe, caused by the spirochete complex *Borrelia burgdorferi* sensu lato [1, 2]. Lyme neuroborreliosis (LNB) is the second most frequent manifestation of LB in children, after the skin manifestation erythema migrans [3]. The incidence of LNB is 28/100,000 in the paediatric population in Sweden [4]. LNB is diagnosed according to European guidelines and requires neurological symptoms suggestive of LNB, pleocytosis in the cerebrospinal fluid (CSF), and intrathecally produced antibodies specific for *B. burgdorferi* [5]. LNB is treated with doxycycline or ceftriaxone according to the guidelines [5]. Among children affected by LNB, 11–25% report persistent symptoms after antibiotic treatment [6–8].

Despite various studies on serum biomarkers in different CNS pathologies, no biomarkers have specifically been identified as prognostic factors of importance for clinical outcomes in children with LNB. Glial fibrillary acidic protein (GFAp), a protein expressed in astrocytes, has been shown to be significantly higher in the CSF of adult LNB patients as compared to healthy controls [9]. However, all adult LNB patients had signs of meningoradiculitis, a symptom relatively uncommon in children with LNB, making a similar study difficult to carry out in a paediatric population. Two biomarkers that have been studied in various clinical settings in both adults and children are neuron-specific enolase (NSE) and S100 calcium-binding protein B (S100B). Prospective analyses of these biomarkers in serum children upon admittance to paediatric intensive care units (PICU) have been shown to predict an unfavourable neurologic outcome in critically ill children with a broad spectrum of admission diagnoses (i.e., neurological disorders, serious infectious diseases, gastrointestinal diseases, and various postoperative complications) [10]. Furthermore, increased concentrations of NSE and S100B have been found in serum, presumably due to blood–brain barrier (BBB) damage, in children with conditions associated with brain damage such as traumatic brain injury [11, 12], in urine in hypoxic-ischemic encephalopathy in asphyxiated full-term infants [13], as well as in CSF in neonatal bacterial meningitis [14].

NSE is a neuron-specific form of the glycolytic enzyme enolase, highly localised to neurons and neuroendocrine cells [15]. NSE is also found in extracerebral cells such as platelets and red blood cells [16, 17]. Increased concentrations of NSE in CSF and serum have been identified in several conditions and diseases, including encephalitis, cerebral infarction, neurodegenerative diseases, and traumatic brain injury [11, 18].

S100B is a member of the S-100 protein family and is produced by astrocytes, oligodendrocytes, and Schwann cells in the CNS [19]. The primary extracerebral sources for S100B are muscle and fat tissue, although S100B has been shown not to correlate with body mass index (BMI), and the extracerebral sources of S100B do not appear to have a significant effect on S100B levels in serum [20]. The serum levels of S100B decrease with increasing age [21], but no significant gender-related differences in serum S100B levels have been shown [21, 22]. Haemolysis is a physiological factor that may interfere with NSE levels in serum, in contrast to S100B, where haemolysis is of no importance to the results of serum levels [22].

There have been problems to establish cut-off values for NSE and S100B in serum in children since values could depend on age, site of extraction, and cohort [23]. However, Bouvier et al. have mapped out reference ranges of serum levels of S100B for children via blood samples from a large cohort of 409 healthy children aged 0–16 years with a reference value of < 0.32 µg/L för children aged 2–16 years [22].

The aim of this study was to investigate whether the brain damage markers NSE and S100B are detectable in serum in paediatric LNB patients and controls and if so, to assess their possible value as prognostic biomarkers for clinical outcomes in children with LNB.

## Material and methods

### Patients and controls

Patients were selected from a previous study of a large cohort of children being evaluated for LNB in central and southeast Sweden during the years 2010–2014 [24]. All patients classified as definite LNB in which serum samples were available were included in this present study as LNB patients (*n* = 61). Children classified as non-LNB patients were included as controls (*n* = 60). Controls were matched for gender and age to the extent made possible by the availability of serum samples.

LNB patients and controls were followed up at 2 months to evaluate clinical recovery as part of the previous prospective study [24]. Based on information from the follow-up visit at a paediatric clinic, including a physical examination and a structured questionnaire for self-reported persistent symptoms (or in some cases, a telephone interview), LNB patients and controls were defined as having complete or incomplete clinical recovery. Clinical characteristics of LNB patients and controls are shown in Table 1.

**Table 1:** Clinical characteristics and laboratory data in LNB patients and controls

	LNB patients (*n* = 61)	Controls (*n* = 60)
Gender female, *n* (%)	26 (43)	27 (45)
Age, median (range)	6 (2–15)	10 (1–17)
Observed tick bite, *n* (%)	37 (61)	28 (47)
Duration of symptoms		
< 1 week, *n* (%)	28 (46)	16 (27)
1–4 weeks, *n* (%)	29 (48)	12 (20)
1–2 months, *n* (%)	1 (2)	4 (7)
> 2 months, *n* (%)	1 (2)	12 (20)
Not specified, *n* (%)	2 (3)	16 (27)
Clinical features on admission		
Facial nerve palsy, *n* (%)	42 (69)	20 (33)
Headache, *n* (%)	43 (70)	40 (67)
Fatigue, *n* (%)	55 (90)	39 (65)
Fever, *n* (%)	31 (51)	12 (20)
Neck pain, *n* (%)	32 (52)	11 (18)
Neck stiffness, *n* (%)	20 (33)	6 (10)
Loss of appetite, *n* (%)	38 (62)	26 (43)
Nausea, *n* (%)	21 (34)	23 (38)
Vertigo, *n* (%)	9 (15)	25 (42)
Erythema migrans, *n* (%)	26 (43)	12 (20)
Laboratory data on admission		
Pleocytosis, median (range)^#^	153 (12–885)	0 (0–4)
Positive *Borrelia* AI*, *n* (%)	61 (100)	0 (0)
Complete clinical recovery at 2-months, *n* (%)	53 (87)	49 (82)

*LNB*, Lyme neuroborreliosis; *n*, number; *#*, total count of leukocytes × 10^6^/L in CSF [25]; *, anti−*Borrelia* IgG and/or IgM antibody index (AI)> 0.3 [26]

### Classification of LNB

LNB was diagnosed according to European guidelines and required neurological symptoms suggestive of LNB without other obvious reasons, pleocytosis in the CSF, and intrathecally produced antibodies specific to *B. burgdorferi* [27]. Non-LNB controls did initially have neurological symptoms suggestive of LNB but had no pleocytosis in the CSF or intrathecally produced antibodies specific to *B. burgdorferi* and did therefore not meet the criteria for LNB. The non-LNB controls consisted of patients with idiopathic facial nerve palsy (*n* = 20), headache (*n* = 35), fatigue UNS (*n* = 2), vertigo (*n* = 1), strabismus (*n* = 1), and paresthesia (*n* = 1). None of the patients in the non-LNB group were diagnosed with any other specific neurological disorder.

### Laboratory methods

Serum samples were drawn from LNB patients and non-LNB controls on admission and stored at − 70 °C. ELISA assays were applied for the detection of NSE and S100B in serum samples retrospectively. In calculating the levels of NSE and S100B in serum from the optical density values of the ELISA assays’ standard curves, a linear regression was used for NSE, and a 4 parametric logistic regression was used for S100B, according to the manufacturers’ instructions.

NSE was analysed with the EDI™ Human Neuron-Specific Enolase ELISA Kit (Epitopic Diagnostics Inc, San Diego, USA), according to the manufacturer’s instructions [28]. Samples from all LNB patients (*n* = 61) and controls (*n* = 60) were available for NSE analysis. Samples with values below the lower detection range (5.0 µg/L) were given half of the value for the lowest standard, at a value of 2.50 μg/L [28]. According to the manufacturer and instructions to users at laboratory units, the reference value for NSE was set at < 16 μg/L [29].

S100B was analysed with the human soluble protein-100B (S100B) ELISA Kit (Cusabio Biotech Co LTD, Wuhan, China), according to the manufacturer’s instructions, with a detection range of 0.078–5 µg/L [30]. Samples available from LNB patients (*n* = 60) and controls (*n* = 59) were analysed for S100B with values below the lower detection range given half of the value for the lowest standard, value 0.039 μg/L. The reference value for S100B (< 0.32 μg/L) was based on previous studies [22].

Intrathecally produced antibodies specific to *B. burgdorferi* (IgG and/or IgM) were analysed as part of the routine diagnostic workup with the IDEIA Lyme Neuroborreliosis Kit according to the manufacturer’s instructions (Oxoid Limited, Hampshire, UK) [31]. The anti-*Borrelia* antibody index (AI) was considered positive if > 0.3 [26]. Pleocytosis was defined as a total count of leukocytes > 5 × 10^6^/L in CSF [25].

In clinical laboratory practice, the standard measure of the BBB function is the ratio between albumin in CSF (mg/L) and albumin in serum (g/L), where an elevated ratio (> 5) demonstrates BBB damage [32]. Data on BBB function, i.e. albumin ratios, were available from the previous study [33] on a number of LNB patients (*n* = 33) and controls (*n* = 45) and could be used in calculations in our present study.

### Statistics

SPSS software was used for statistical calculations. The chi-squared test and Fisher’s exact test were used for dichotomous data, and Mann–Whitney *U* test was used for continuous nonparametric data. Spearman’s test was used in calculating correlations. A *p*-value < 0.05 was considered statistically significant.

### Ethical considerations

The study was approved by the Regional Ethics Committee in Uppsala, Sweden (Dnr. 2010/106). Informed written consent was received from patients/guardians.

## Results

The brain damage marker NSE was detectable in 115 out of 121 (95%) serum samples drawn on admission from LNB patients and controls. NSE concentrations in serum were elevated above the reference value (16 μg/L) in 38 of 61 (62%) LNB patients and in 31 of 60 (52%) controls. No significant differences in NSE concentrations were found when comparing LNB patients and controls (Table 2).

**Table 2:** NSE, S100B, and Albumin ratios (CSF/serum) in LNB patients and controls

	LNB patients	Controls	*P*-value
NSE μg/L, median (range)	19.21 (8.25–70.47)	17.01 (2.50–393.28)	0.165
S100B μg/L, median (range)	0.04 (0.04–0.79)	0.04 (0.04–0.21)	0.908
NSE above cut-off (16 μg/L), *n* (%)	38 (62%)	31 (52%)	0.273
S100B above cut-off (0.32 μg/L), *n* (%)	3 (5%)	0 (0%)	0.244
Albumin in CSF mg/L, median (range)	383 (114–839)	99.4 (49.4–291)	** < **0.001
Albumin in serum g/L, median (range)	40.5 (35.3–48.7)	40.3 (31.1–46.6)	0.856
Albumin ratio (CSF/serum), median (range)	8.86 (2.75–20.39)	2.61 (1.23–7.13)	** < **0.001
BBB-damage, *n* (%)	27 (82)	3 (7)	** <** 0.001

*LNB*, Lyme neuroborreliosis; *NSE*, neuron-specific enolase; *n*, number; *S100B*, S100 calcium-binding protein B; *CSF*, cerebrospinal fluid; BBB (blood–brain barrier) damage = CSF/serum albumin ratio > 5 [32]

The brain damage marker S100B was detectable in 12 out of 119 (10%) available serum samples drawn on admission from LNB patients and controls. S100B concentrations were elevated above the reference value (0.32 μg/L) in 3 of 60 (5%) LNB patients and 0 of 59 (0%) controls. No significant differences in S100B concentrations were found in LNB patients as compared to controls (Table 2).

No positive or negative correlations were found when comparing age and concentration of NSE (rho =  − 0.157, *p* = 0.086) and S100B (rho = 0.035, *p* = 0.703) in LNB patients and controls (data not shown). Nor did we find any correlations between the duration of symptoms on admission and levels of NSE (rho =  − 0.022, *p* = 0.808) or S100B (rho = 0.085, *p* = 0.355) in serum (data not shown).

Among LNB patients, 53 out of 61 (87%) showed a complete clinical recovery, and among controls, 49 out of 60 (82%) showed a complete clinical recovery at the 2-month follow-up. Among LNB patients, there were no significant differences in NSE and S100B concentrations in serum when comparing patients with complete and incomplete clinical recovery at the 2-month follow-up (*p* = 0.571 and *p* = 0.321, respectively) (data not shown).

**Fig. 1:**
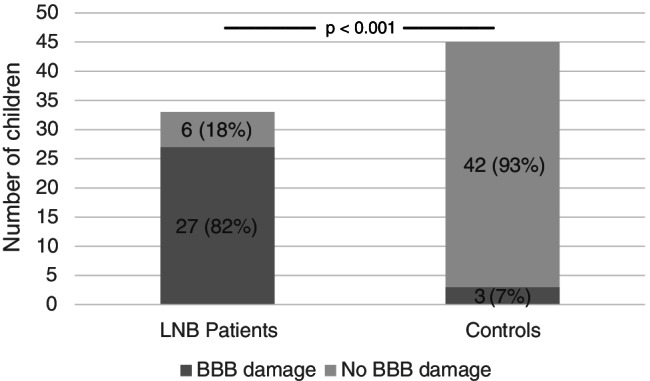
Blood–brain barrier (BBB) damage in patients with Lyme neuroborreliosis (LNB) and controls (*p* < 0.001)

Data on albumin ratio (CSF/serum) was available for evaluation in 33 out of 61 (54%) LNB patients and in 45 out of 60 (75%) controls. BBB damage (CSF/serum albumin ratio > 5) was found in 82% of LNB patients and in 7% of controls (*p* < 0.001) (Fig. 1), and the ratio itself was significantly higher in LNB patients as compared to controls (*p* < 0.001) (Table 2).

**Fig. 2:**
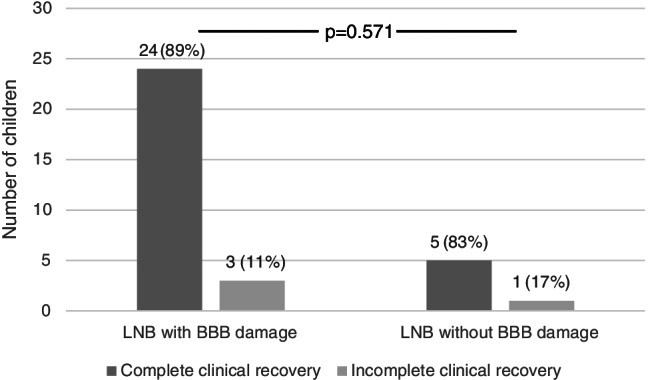
Clinical recovery in patients with Lyme neuroborreliosis (LNB), with or without blood–brain barrier (BBB) damage (*p* = 0.571)

Complete clinical recovery was observed in 24 out of 27 (89%) LNB patients with BBB damage and in 5 out of 6 (83%) LNB patients without BBB damage (*p* = 0.571) (Fig. 2). Among controls with BBB damage, complete clinical recovery was observed in all three patients, and 36 out of 42 (86%) controls without BBB damage (*p* = 0.644) (data not shown).

NSE levels in serum were elevated above cut-off in 16 out of 27 (60%) LNB patients with BBB damage and in 5 out of 6 (83%) LNB patients without BBB damage. The median concentration of NSE in serum did not differ between LNB patients with or without BBB damage (*p* = 0.424) (data not shown), nor did the median concentration of S100B (*p* = 0.451) (data not shown).

## Discussion

In this study, it is shown that levels of NSE and S100B in serum on admission were not significantly higher in LNB patients as compared to non-LNB controls. To our knowledge, there are only a few studies on brain damage markers in LNB patients, and no significant biomarkers for clinical recovery have yet been identified. A previous study showed detectable levels of the brain damage markers NSE, S100B, GFAp, and neurofilament protein (NFL) in CSF in adult LNB patients before antibiotic treatment [34], suggesting that LNB may affect CNS parenchyma. However, that study is not comparable to our present study since the markers were analysed in CSF as opposed to serum. In our present study, most LNB patients had BBB damage, which should allow for brain damage markers to pass from CSF to serum. However, relatively few LNB patients showed elevated brain damage markers in serum (above cut-off), which is somewhat unexpected. It could possibly be explained by paediatric LNB patients often having a milder CNS involvement with a shorter duration of symptoms than adult LNB patients, or by the fact that the cut-off levels for the brain damage markers may vary with age, as reported in several previous studies [21, 22, 35, 36]. However, we found no correlation between the concentration of NSE or S100B and age. In addition, in the present study, only a few patients were under the age of 2 years, the age group in which the strongest negative correlation between age and S100B was seen in previous studies [22, 35, 36].

In our present study, there were no significant differences in the levels of NSE and S100B in serum on admission in LNB patients with complete or incomplete clinical recovery at the 2-month follow-up. This is in line with an earlier study that showed that the pretreatment levels of NSE and S100 proteins in CSF were not significantly higher in adult LNB patients with sequelae [34]. Thus, our results confirm that NSE and S100B in serum could not be useful as prognostic biomarkers for clinical outcomes.

Dotevall et al. compared pre- and posttreatment levels of brain damage markers NSE, S100B, GFAp, and neurofilament protein (NFL) in CSF in adult LNB patients and found that all four brain damage markers were reduced in CSF after treatment, indicating an improvement of CNS impairment [34]. In our paediatric LNB patients, data on posttreatment levels of NSE and S100B in serum or CSF were unfortunately not available. However, most patients were assessed as being recovered at the 2-month follow-up as an indication of relevant post treatment clinical improvement.

Admittedly, there are limitations to our study. The ELISA assays on NSE and S100B were conducted with single sample testing, where duplicates would have given more reliable data, as always when performing laboratory testing. However, since we found no differences when comparing LNB patients and controls with nonparametric statistics, this limitation should not have had a large negative impact on our results.

Another possible weakness of our present study is that most of the analysed serum samples had an S100B concentration below the lower detection range. When choosing from the several different ELISA test kits available, we based our choice on a kit with a reasonably wide detection range (0.078–5.0 µg/L), which also included previously documented levels of S100B in healthy children [22]. Admittedly, it could have been of interest to investigate even lower levels of S100B in pediatric LNB, but as most of our results were considerably lower than the reference value of 0.32 µg/L, the relevance may be questioned.

Our control group consisted of children with symptoms initially suggestive of neuroborreliosis but demonstrated neither pleocytosis nor intrathecally produced antibodies for *B. burgdorferi* and, as such, functioned as non-LNB controls. Theoretically, this group may have contained patients with very early LNB before any findings in CSF materialised, but this would arguably be true in only a few controls and thus presumably have a negligible effect on our results. A second control group of healthy children without any neurological symptoms suggestive of LNB would potentially have added important information to our findings, but we nonetheless consider our control group very relevant in the clinical context. The group represents children that undergo lumbar punctures according to common practice in cases where LNB is suspected but, despite their symptoms, have negative CSF results.

At the 2-month follow-up, a few patients (and/or their guardian) were interviewed via telephone without a physical appointment and therefore did not undergo a physical examination by a paediatrician. This is a weakness of the study, which might have influenced the evaluation of clinical recovery in a few cases. However, the follow-up always included a structured questionnaire for self/parent-reported symptoms (patient-reported outcome measures or PROMs), which is the most important measurement of clinical recovery. Consequently, we find it highly probable that the LNB patients in our study were correctly assessed as having complete or incomplete clinical recovery at the 2-month follow-up. Thus, our results that serum levels of NSE and S100B did not differ between LNB patients with complete or incomplete clinical recovery can be considered reliable, and therefore we deduce that these two brain damage markers, at least when analyzed in serum, are not useful for prognostic purposes.

## Conclusion

The brain damage marker NSE was detectable in serum samples from a majority of LNB patients and controls, but S100B was detectable only in a few LNB patients and none of the controls. NSE and S100B concentrations did not differ significantly in LNB patients as compared to controls, nor were they significantly elevated when comparing LNB patients with complete and incomplete clinical recovery at the 2-month follow-up. Thus, NSE and S100B in serum could not be recommended as prognostic biomarkers for clinical outcomes in children with LNB.

## Data Availability

The data that support the findings of this study are available from the corresponding author upon reasonable request, provided that the data can be made available in accordance with applicable data protection and privacy regulations.

## Abbreviations

BBB: Blood-brain barrier
BMI: Body mass index
CNS: Central nervous system
CSF: Cerebrospinal fluid
ELISA: Enzyme-linked immunosorbent assay
GFAp: Glial fibrillary acidic protein
LB: Lyme borreliosis
LNB: Lyme neuroborreliosis
*n*: Number
NSE: Neuron-specific enolase
S100B: S100 calcium-binding protein B
